# Network Pharmacology Strategy to Investigate the Pharmacological Mechanism of HuangQiXiXin Decoction on Cough Variant Asthma and Evidence-Based Medicine Approach Validation

**DOI:** 10.1155/2020/3829092

**Published:** 2020-10-30

**Authors:** Qingqing Xia, Mingtao Liu, Hui Li, Lijun Tian, Jia Qi, Yufeng Zhang

**Affiliations:** ^1^Department of Respiratory Medicine, Jiangyin Hospital of Traditional Chinese Medicine, Jiangyin Hospital Affiliated to Nanjing University of Chinese Medicine, Jiangyin, Jiangsu 214400, China; ^2^Department of Respiratory Medicine, Binzhou People's Hospital, Binzhou, Shandong 256610, China; ^3^Department of Critical Care Medicine, Nantong Third People's Hospital, Nantong University, Nantong, Jiangsu 226001, China; ^4^Department of Pharmacy, Xin Hua Hospital Affiliated to Shanghai Jiao Tong University School of Medicine, Shanghai 200092, China

## Abstract

**Objective:**

To investigate the pharmacological mechanism of HuangQiXiXin decoction (HQXXD) on cough variant asthma (CVA) and validate the clinical curative effect.

**Methods:**

The active compounds and target genes of HQXXD were searched using TCMSP. CVA-related target genes were obtained using the GeneCards database. The active target genes of HQXXD were compared with the CVA-related target genes to identify candidate target genes of HQXXD acting on CVA. A medicine-compound-target network was constructed using Cytoscape 3.6.0 software, and a protein-protein interaction (PPI) network was constructed using the STRING database. Gene ontology (GO) function enrichment and Kyoto Encyclopedia of Genes and Genomes (KEGG) pathway enrichment analysis were performed using RGUI3.6.1 and Cytoscape 3.6.0. We searched the main database for randomized controlled trials of HQXXD for CVA. We assessed the quality of the included studies using the Cochrane Reviewers' Handbook. A meta-analysis of the clinical curative effect of HQXXD for CVA was conducted using the Cochrane Collaboration's RevMan 5.3 software.

**Results:**

We screened out 48 active compounds and 217 active target genes of HQXXD from TCMSP. The 217 active target genes of HQXXD were compared with the 1481 CVA-related target genes, and 132 candidate target genes for HQXXD acting on CVA were identified. The medicine-compound-target network and PPI network were constructed, and the key compounds and key targets were selected. GO function enrichment and KEGG pathway enrichment analysis were performed. Meta-analysis showed that the total effective rate of the clinical curative effect was significantly higher in the experimental group than the control group.

**Conclusion:**

The pharmacological mechanism of HQXXD acting on CVA has been further determined, and the clinical curative effect of HQXXD on CVA is remarkable.

## 1. Introduction

Cough variant asthma (CVA) has the same pathogenesis as asthma and is characterized by persistent chronic airway inflammation and airway hyperresponsiveness [1]. The clinical manifestations of CVA are chronic cough, which is not always accompanied by wheezing. Chronic cough is often the only symptom of CVA [2]. CVA is the most common cause of chronic cough in China [3] and the second most common cause of chronic cough in Korea [4].

Inhalation of corticosteroids and long-acting *β*2-agonists is recommended for the treatment of CVA [5, 6]. However, there is a high recurrence rate of the disease, and the long-term effect of these treatments is not ideal. Recently, Chinese herbal medicine has been used in the treatment of CVA [7, 8]. HuangQiXiXin decoction (HQXXD), added or reduced from the traditional Chinese medicine (TCM) prescriptions Yupingfeng powder and Zhisou powder, mainly consists of Huangqi, Xixin, Jingjie, Fangfeng, Huangqin, Baizhu, Fuling, Chantui, Banxia, Baibu, and Gancao. It has been reported that HQXXD is effective in the treatment of CVA; however, our previous study showed that the total effective rate of HQXXD in the treatment of CVA was not significant compared with the control group, although there was some evidence of a clinical effect [9].

In this study, we used network pharmacology and an evidence-based medicine approach to determine the pharmacological mechanism of HQXXD on CVA and validate the clinical curative effect. First, we identified the active compounds and target genes of the monarch and minister herbs in HQXXD. Next, we used the CVA-related target genes and the active target genes of HQXXD to construct the medicine-compound-target network and protein-protein interaction (PPI) network. Subsequently, gene ontology (GO) function enrichment and Kyoto Encyclopedia of Genes and Genomes (KEGG) pathway enrichment were performed. Finally, we screened randomized controlled trials (RCTs) that investigated the clinical curative effect of HQXXD on CVA and performed a meta-analysis to validate the clinical curative effect.

## 2. Materials and Methods

### 2.1. Screening of Active Compounds in HQXXD

Huangqi, Xixin, Jingjie, and Fangfeng are monarch and minister herbs, which are regarded as the main active herbs in HQXXD, and have the Latin names Radix Astragali (RA), Herba cum Radix Asari (HRA), Herba Schizonepetae (HS), and Radix Saposhnikoviae (RS), respectively. We have determined that these four herbs are the main components in HQXXD. The compounds in these four herbs were obtained using the Traditional Chinese Medicine Systems Pharmacology Database and Analysis Platform (TCMSP) (http://tcmspw.com/tcmsp.php) [10]. We used the parameters of oral bioavailability (OB) ≥ 30% and drug-likeness (DL) ≥ 0.18 to screen for active compounds, and OB and DL are commonly used in network pharmacology. OB relates to the rate and extent of which the active component or components of the drug are absorbed into the body. DL indicates the degree to which a compound contains specific functional groups or has the same or similar physical characteristics to most drugs.

### 2.2. Screening of the Target Genes of Active Compounds

We retrieved the corresponding target genes of active compounds from TCMSP. After importing the target genes into the UniProt knowledgebase (http://www.uniprot.org/) and setting the search format as “Homo sapiens,” the human official gene symbols were identified, and the active target genes of HQXXD were screened out.

### 2.3. Acquisition of CVA-Related Target Genes and Identification of Candidate Target Genes of HQXXD Acting on CVA

Using “cough variant asthma” as the keyword in the GeneCards database (https://www.genecards.org/), we searched and acquired CVA-related target genes. Then, the active target genes of HQXXD were compared with the CVA-related target genes, and the intersecting genes were defined as the candidate target genes for HQXXD acting on CVA.

### 2.4. Construction of the Medicine-Compound-Target Network and Selection of Key Compounds

We used Cytoscape 3.6.0 software and its Network Analyzer tool function to construct and analyze a medicine-compound-target network [11]. Nodes represented compounds and target genes, and the relationships between them were represented as edges. The key compounds in HQXXD acting on CVA were selected according to the degree of connection between the compound and the target gene.

### 2.5. Construction of the PPI Network and Selection of Key Targets

The candidate target genes were introduced into the STRING database (https://string-db.org/) to construct a PPI network of HQXXD acting on CVA. The research species was defined as “Homo sapiens,” the lowest interaction score was set to 0.4, and the rest of the parameters were set to the default settings to obtain the PPI network. Using Cytoscape 3.6.0 software and its Network Analyzer tool, the PPI network was subjected to topology analysis, and the key targets of HQXXD acting on CVA were then selected according to the degree values of each target gene in the PPI network.

### 2.6. GO Function Enrichment and KEGG Pathway Enrichment Analysis

RGUI3.6.1 and org.Hs.eg.db package were applied to obtain the Entrez IDs of candidate target genes. Then, we used RGUI, the clusterProfiler package, and Cytoscape to perform GO function enrichment analysis, which included biological process (BP), molecular function (MF), and cellular component (CC) analysis, and KEGG pathway enrichment analysis [12]. Adjusted *P* < 0.05 was considered as statistically significant.

### 2.7. Validation of the Clinical Curative Effect of HQXXD on CVA

#### 2.7.1. Screening of RCTs Investigating the Clinical Curative Effect of HQXXD on CVA

We searched using the terms “HuangQiXiXin decoction” and “cough variant asthma” in PubMed, the Cochrane Central Register of Controlled Trials, the Chinese Biomedical Literature database, Chinese National Knowledge Infrastructure (CNKI), the Chongqing VIP database (CQVIP), and Wanfang Data from the establishment of each database to January 15, 2020. When the terms were not included in the titles and abstracts, if the terms were present in the full text, the full-text paper was also screened.

The inclusion criteria included that the study was designed as a RCT, the participants had symptoms that were in accordance with a diagnosis of CVA, HQXXD was used in the experimental group, the control group used conventional therapy without TCM therapy, and the clinical curative effect was the main outcome measure. Exclusion criteria included studies where there were incomplete data on the clinical curative effect. For reports of the same study, the earliest published report was included and the rest were excluded.

#### 2.7.2. Data Extraction and Quality Assessment

Two reviewers independently extracted the information from the included studies. The main information included the first author, year of publication, number of patients in each group, methods of intervention in the experimental and control groups, and outcome data of the clinical curative effect.

We used the Cochrane Reviewers' Handbook for guidelines to assess the risk of bias. Seven criteria were used: random sequence generation; allocation concealment; patient blinding; assessor blinding; incomplete outcome data; selective outcome reporting; and other risks of bias [13].

These main data were input into the Cochrane Collaboration's RevMan 5.3 software for meta-analysis to analyze the clinical efficacy of HQXXD on CVA.

### 2.8. Statistical Analysis

Some of the statistical analyses were performed automatically using the bioinformatic tools of the platforms and software mentioned above. In GO function enrichment and KEGG pathway enrichment, adjusted *P* < 0.05 was considered as statistically significant. When RevMan 5.3 software was used for meta-analysis, the total efficiency rates were expressed as the odds ratio (OR) with 95% confidence interval (CI). Heterogeneity was assessed by the *Q* test (*P* value and *I*^2^), and *P* < 0.10 indicated heterogeneity across studies. Studies with *I*^2^ < 50% were considered to have no heterogeneity and those with *I*^2^ ≥ 50% were considered to have heterogeneity. If no heterogeneity was detected, the fixed effects model was used as the pooling method; otherwise, the random effect model was considered to be the appropriate choice [14, 15]. *P* < 0.05 was considered as statistically significant.

## 3. Results

### 3.1. Screened Active Compounds in HQXXD

We obtained 87 compounds from RA, 192 compounds from HRA, 159 compounds from HS, and 173 compounds from RS in TCMSP (Supplementary files 1A, B, C, and D). Setting the conditions as OB ≥ 30% and DL ≥ 0.18, 20 active compounds from RA, 8 active compounds from HRA, 11 active compounds from HS, and 18 active compounds from RS were selected. Finally, with exclusion of duplications, 53 active compounds from HQXXD were identified. The basic information on the active compounds from HQXXD is shown in Table 1.

### 3.2. Screened Active Target Genes of HQXXD

The corresponding target genes of the 53 active compounds were also obtained in TCMSP (Supplementary file 2A). Using the UniProt knowledgebase, the corresponding gene symbols were screened by setting the format as “Homo sapiens” (Supplementary file 2B (available here)). Four compounds (MOL000374, MOL000398, MOL000438, and MOL001506) did not have targets, and one compound (MOL000439) did not have a compatible gene symbol. Finally, 217 active target genes of 48 active compounds from HQXXD were identified (Supplementary file 2C).

### 3.3. Acquired CVA-Related Target Genes and Identified Candidate Target Genes of HQXXD Acting on CVA

Using “cough variant asthma” as the keyword in the GeneCards database, 1481 CVA-related target genes were acquired (Supplementary files 3A, B).

**Figure 1:**
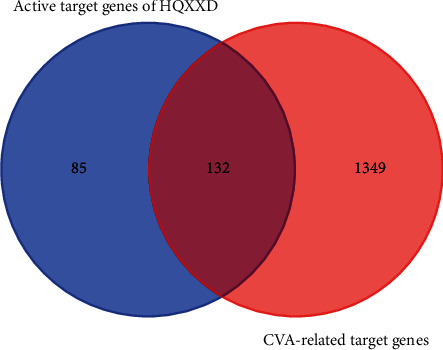
Candidate target genes of HQXXD acting on CVA. The 217 active target genes of HQXXD were compared with the 1481 CVA-related target genes; then, 132 candidate target genes of HQXXD acting on CVA were identified.

The 217 active target genes of HQXXD were compared with the 1481 CVA-related target genes, and 132 candidate target genes of HQXXD acting on CVA were identified (Figure 1, Table 2).

### 3.4. Constructed Medicine-Compound-Target Network and Selected Key Compounds

The 132 overlapping candidate target genes corresponded to 47 active compounds (MOL000433 had correspondence to an overlapping candidate target gene), including 15 compounds from RA, 8 compounds from HRA, 10 compounds from HS, and 16 compounds from RS (Supplementary file 4).

**Figure 2:**
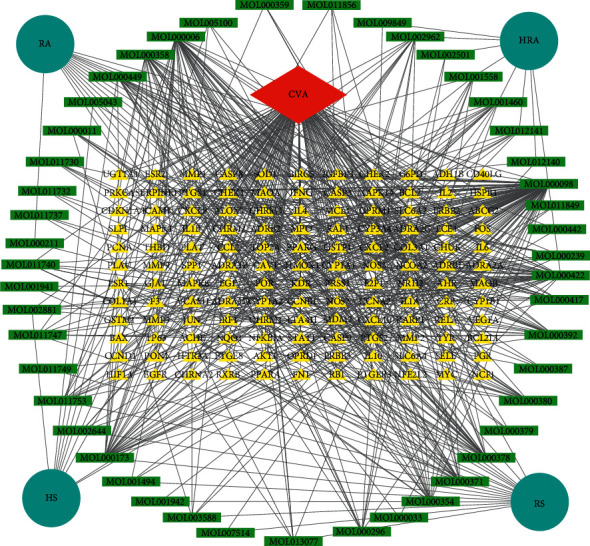
Medicine-compound-target network. There were 184 nodes (4 medicine nodes, 47 compound nodes, 132 target gene nodes, and 1 CVA node) and 653 edges in the network.

Then, a network of medicine-compound-target was constructed using the Cytoscape software and analyzed by the Network Analyzer tool. There were 184 nodes (4 medicine nodes, 47 compound nodes, 132 target gene nodes, and 1 CVA node) and 653 edges in the network (Figure 2). The top 14 compounds ranked by degree in the network were MOL000098, MOL000006, MOL000422, MOL000173, MOL000378, MOL000358, MOL000392, MOL000371, MOL000449, MOL000354, MOL000380, MOL000417, MOL001460, and MOL002962 (Table 3), which could be considered the key compounds in HQXXD acting on CVA.

### 3.5. Constructed PPI Network and Selected Key Targets

**Figure 3:**
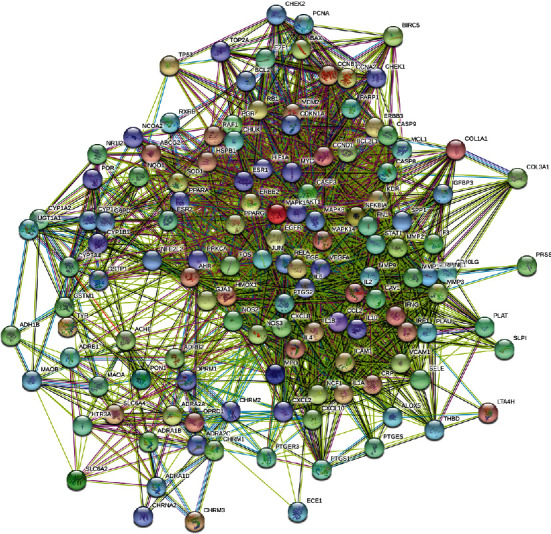
The PPI network of HQXXD acting on CVA. When the lowest interaction score was set to 0.40, 132 target proteins in the network had an interaction and 2373 edges represented the interactions between the proteins.

To further study the mechanism of HQXXD acting on CVA, 132 overlapping target genes were mapped into the STRING database, and the PPI network was obtained. When we set the lowest interaction score to 0.40, 132 target proteins in the network had interactions, and 2373 edges represented the interactions between the proteins (Supplementary file 5, Figure 3). The top 20 target genes ranked by degree in the network are shown in Table 4, which can be considered the key targets of HQXXD acting on CVA.

### 3.6. GO Function Enrichment and KEGG Pathway Enrichment Analysis

The Entrez IDs of candidate target genes were obtained using RGUI and org.Hs.eg.db (Table 2). Then, GO function enrichment and KEGG pathway enrichment analysis were performed using RGUI and clusterProfiler.

**Figure 4:**
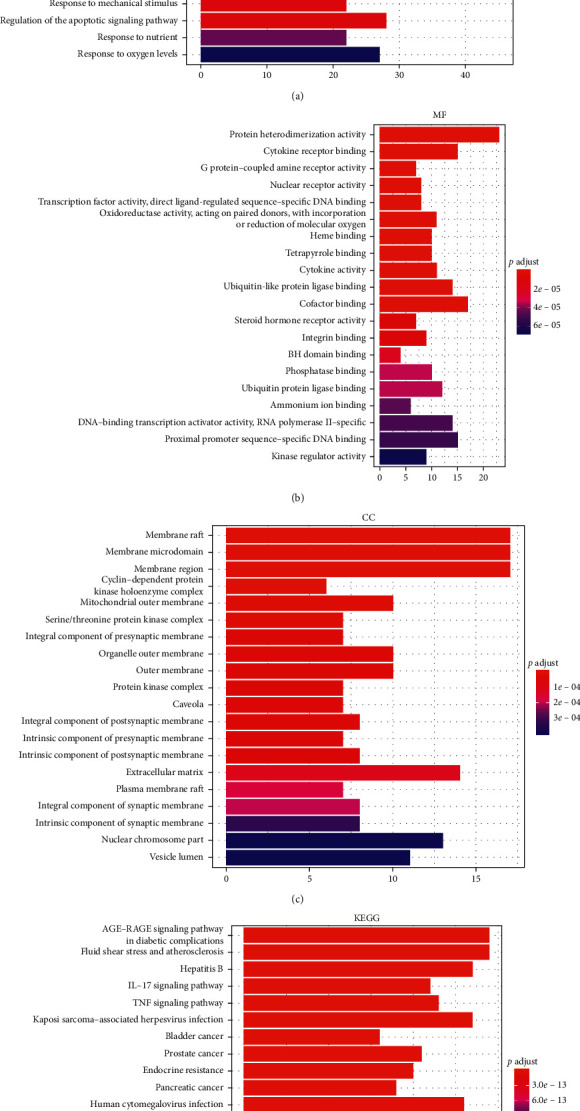
Top 20 GO function and KEGG pathway enrichments. (a) The top 20 enriched BP functions of candidate target genes; (b) the top 20 enriched MF activities of candidate target genes; (c) the top 20 enriched CC regions of candidate target genes; and (d) the top 20 KEGG pathway enrichments.

GO BP function enrichment analysis showed that the candidate target genes of HQXXD acting on CVA were significantly enriched in response to toxic substance, response to molecule of bacterial origin, response to lipopolysaccharide, response to extracellular stimulus, response to nutrient levels, response to metal ion, response to reactive oxygen species, response to oxidative stress, cellular response to oxidative stress, response to antibiotic, and so on (Supplementary file 6A). The top 20 GO BP enrichments ranked by the adjusted *P* value are shown in Figure 4(a).

GO MF function enrichment analysis showed that the candidate target genes of HQXXD acting on CVA that were significantly enriched in protein heterodimerization activity, cytokine receptor binding, *G* protein-coupled amine receptor activity, nuclear receptor activity, transcription factor activity direct ligand-regulated sequence-specific DNA binding, oxidoreductase activity acting on paired donors with incorporation or reduction of molecular oxygen, heme binding, tetrapyrrole binding, cytokine activity, ubiquitin-like protein ligase binding, and so on (Supplementary file 6B). The top 20 GO MF enrichments ranked by the adjusted *P* value are shown in Figure 4(b).

GO CC function enrichment analysis showed that candidate target genes of HQXXD acting on CVA were significantly enriched in membrane raft, membrane microdomain, membrane region, cyclin-dependent protein kinase holoenzyme complex, mitochondrial outer membrane, serine/threonine protein kinase complex, integral component of presynaptic membrane, organelle outer membrane, outer membrane, protein kinase complex, and so on (Supplementary file 6C). The top 20 GO CC enrichments ranked by the adjusted *P* value are shown in Figure 4(c).

KEGG pathway enrichment analysis was further conducted for determining candidate target genes of HQXXD acting on CVA. Candidate target genes were significantly enriched in advanced glycation end products- (AGE-) receptor for the AGE (RAGE) signaling pathway in diabetic complications, fluid shear stress and atherosclerosis, hepatitis B, interleukin- (IL-) 17 signaling pathway, tumor necrosis factor (TNF) signaling pathway, Kaposi sarcoma-associated herpesvirus infection, bladder cancer, prostate cancer, endocrine resistance, pancreatic cancer, and so on (Supplementary file 6D). The top 20 KEGG pathway enrichments ranked by the adjusted *P* value are shown in Figure 4(d).

**Figure 5:**
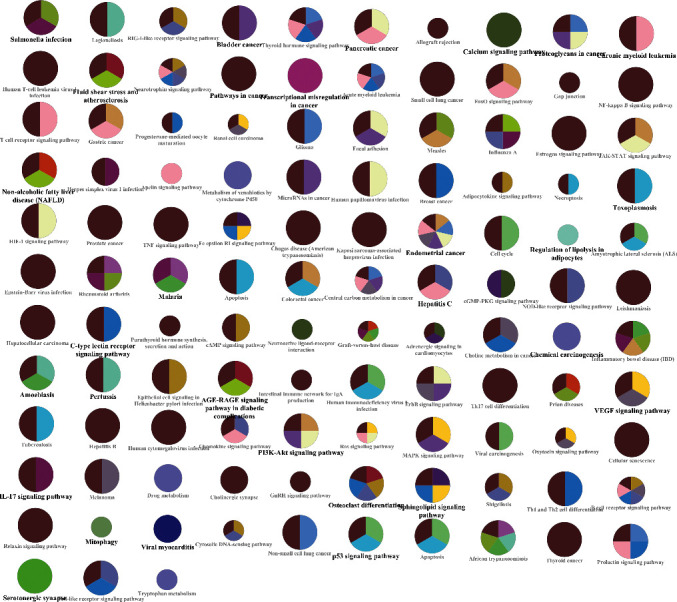
GO function enrichment and KEGG pathway enrichment from ClueGO.

Using the ClueGO plugin app and CluePedia plugin app of the Cytoscape software, the GO function enrichment and KEGG pathway enrichment from ClueGO can be displayed more intuitively as shown in Figure 5.

### 3.7. Validation of the Clinical Curative Effect of HQXXD on CVA

#### 3.7.1. Screened RCTs Investigating the Clinical Curative Effect of HQXXD on CVA

**Figure 6:**
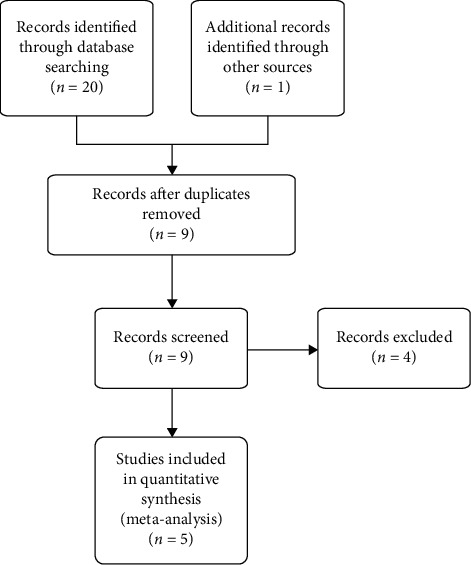
Flow chart of the research selection process. A total of 21 studies were retrieved through database searching. After removing duplication, nine studies were retained. A total of four irrelevant studies were excluded after reading the title, abstract, and full text. Five RCTs were included for further evaluation.

A total of 21 studies were retrieved through database searching. After removing duplication, nine studies were retained. A total of four irrelevant studies were excluded after reading the title, abstract, and full text. Five RCTs [16–20] were included for further evaluation. The literature screening process and results are shown in Figure 6.

#### 3.7.2. Description of Included RCTs and Assessment of the Methodological Quality

Five eligible RCTs were identified. The five RCTs were all conducted in China and included 342 patients. The five studies were all single-center studies. The basic features of the included studies are outlined in Table 5.

**Figure 7:**
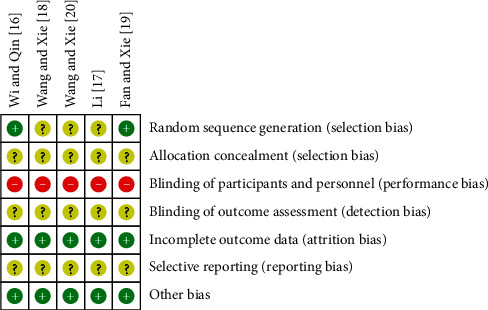
Risk of bias summary. Review authors' judgments about each risk of bias item for each included study.

Two RCTs [16, 19] employed adequate methods of random sequence generation; none of the RCTs introduced allocation concealment; none of the RCTs described blindness; all the RCTs had complete outcome data; and for all studies, we were unable to determine if these were selective reports (Figure 7).

#### 3.7.3. Meta-Analysis

**Figure 8:**
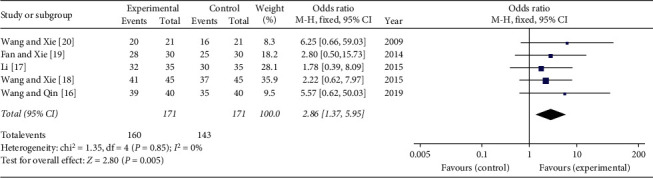
Forest plot of comparison: total effective rate of the clinical curative effect. When the fixed effect model was used to merge OR values, the pooled OR was 2.86 (95% CI 1.37–5.95, *Z* = 2.80, *P*=0.005). This result indicated that the total effective rate of the clinical curative effect was significantly higher in the experimental group compared with the control group.

The five studies that compared the total effective rate of the clinical curative effect included a total of 342 participants, 171 in the experimental groups and 171 in the control groups. The five studies had homogeneity (heterozygosity test, Chi^2^ = 1.35, *P*=0.85, *I*^2^ = 0%). When the fixed effect model was used to merge OR values, the pooled OR was 2.86 (95% CI 1.37–5.95, *Z* = 2.80, *P*=0.005). This indicated that the total effective rate of the clinical curative effect was significantly higher in the experimental group compared with the control group (Figure 8).

## 4. Discussion

CVA is characterized by wheezing cough, wind cough, and stubborn cough in TCM, which attributes qi deficiency and vigorous wind as the main causes of this disease and emphasizes that the treatment of patients should be based on this aspect [21].

HQXXD is added or reduced from the TCM prescription Yupingfeng powder and Zhisou powder [9]. RA, HRA, HS, and RS are monarch and minister herbs in HQXXD, which are regarded as the main active herbs in HQXXD. RA is believed to tonify lung qi; HRA, HS, and RS are believed to tonify the spleen and stomach and dispel wind dampness, which strengthen the effect of RA.

Chinese herbal medicines are complex and contain many compounds. At present, most studies of TCM are limited to explaining the mechanism of a certain compound and lack a holistic view. Currently, network pharmacology is widely used in the study of TCM and advocates whole and group analysis, which is consistent with the multicomponent, multitarget, and multipathway characteristics of TCM [22–24].

We identified the active compounds and target genes of HQXXD from TCMSP. Then, 132 candidate target genes of HQXXD acting on CVA were identified, and the key compounds of HQXXD acting on CVA were selected. The main key compounds were quercetin, luteolin, kaempferol, wogonin, 7-*O*-methylisomucronulatol, beta-sitosterol, formononetin, 3,9-di-*O*-methylnissolin, stigmasterol, and isorhamnetin. Quercetin has a potential protective and preventive effect on the frequency of attacks and in controlling the most common symptoms of asthma [25]; quercetin has both immunomodulatory and bronchodilatory properties [26]. Luteolin has been shown to have an antiallergic effect in a murine model of allergic asthma and rhinitis [27]. Kaempferol may be a potent antiallergic compound targeting the allergic asthma typical of airway hyperplasia and hypertrophy [28]; kaempferol could suppress eosinophil infiltration and airway inflammation in airway epithelial cells and in mice with allergic asthma [29]. Wogonin could attenuate ovalbumin-induced airway inflammation in a mouse model of asthma [30]. Beta-sitosterol possesses antiasthmatic actions by inhibiting the cellular responses and subsequent release/synthesis of Th2 cytokines [31]. Stigmasterol has antiasthmatic properties and suppressive effects on the key features of allergen-induced asthma [32]. The results of our study are in accordance with these previous studies, which indicate that HQXXD has an effect on CVA because of the action of these multiple compounds.

PPI network analysis suggested that the action of HQXXD on CVA is related to multiple targets. The key targets were AKT1, IL6, VEGFA, JUN, EGFR, CXCL8, CASP3, MMP9, PTGS2, and MAPK8. Previous research has indicated that AKT1 is related to pathway mechanisms in children with severe asthma [33]; IL6 polymorphisms are related to the effects of smoking on the risk of adult asthma [34]; VEGFA rs833069 SNPs are associated with asthma [35]; VEGFA is associated with the response to inhaled corticosteroids in children with asthma [36]; smoking aggravates airway inflammation by activating the *c*-Jun amino terminal kinase pathway in asthma [37]; EGFR activation-induced decreases in claudin 1 promote MUC5AC expression and exacerbated asthma in mice [38]; the EGRF-dependent signaling pathway is associated with diseases, such as asthma [39]; neutrophils release CXCL8, NE, and MMP-9 in response to viral surrogates with R848-induced CXCL8 release being specifically enhanced in neutrophils in asthma [40]. CASP3 may play a role in allergic asthma [41]; the PTGS2 gene is associated with diisocyanate-induced asthma [42]. The relationships between these key targets and CVA, some of which have been well studied, could be further studied, and possible mechanisms that have not been previously studied could be explored.

GO functional enrichment analysis suggested that GO BP for HQXXD in the treatment of CVA were enriched in response to toxic substances, molecules of bacterial origin, lipopolysaccharides, extracellular stimuli, nutrient levels, metal ions, reactive oxygen species, oxidative stress, antibiotics, and cellular responses to oxidative stress. GO MF analysis indicated that HQXXD in the treatment of CVA involved in protein heterodimerization activity, cytokine receptor binding, *G* protein-coupled amine receptor activity, nuclear receptor activity, transcription factor activity, direct ligand-regulated sequence-specific DNA binding, oxidoreductase activity acting on paired donors with incorporation or reduction of molecular oxygen, heme binding, tetrapyrrole binding, cytokine activity, and ubiquitin-like protein ligase binding. GO CC analysis indicated that HQXXD in the treatment of CVA were enriched in the membrane raft, membrane microdomain, membrane region, cyclin-dependent protein kinase holoenzyme complex, mitochondrial outer membrane, serine/threonine protein kinase complex, integral component of presynaptic membrane, organelle outer membrane, outer membrane, and protein kinase complex. These regions are closely related to airway inflammation, airway epithelial cell injury, airway hyperresponsiveness, and airway remodeling, which are closely related to the pathological mechanisms of CVA [43–45].

KEGG enrichment pathway analysis showed that many pathways were closely related to the pathogenesis of CVA. The main pathways included the AGE-RAGE signaling pathway in diabetic complications, fluid shear stress and atherosclerosis, the IL-17 signaling pathway, TNF signaling pathway, and pathways involved in hepatitis B, Kaposi sarcoma-associated herpes virus infection, bladder cancer, prostate cancer, endocrine resistance, and pancreatic cancer. A relationship between these pathways and CVA has also been found or preliminarily indicated. These results indicated that HQXXD acts on CVA through multiple pathways. The main pathways and main target genes in the pathways are worthy of further study.

The pharmacological mechanisms of HQXXD acting on CVA have been investigated through a network pharmacology approach, but there are some limitations with this approach. We identified active compounds and target genes of HQXXD from the TCMSP database and acquired CVA-related target genes from the GeneCards database. Although these databases are the most comprehensive databases currently available, the data collection is still not necessarily comprehensive, and the establishment of the screening criteria for active compounds cannot be completely accurate. In addition, we constructed a PPI network and performed GO function enrichment and the KEGG pathway enrichment analysis to investigate the pharmacological mechanisms of HQXXD acting on CVA. Further investigation of the key target genes and pathways through experimental analysis is required, which will be the focus of future research in our laboratory.

In addition, we used an evidence-based medicine approach to perform a meta-analysis of the clinical curative effect of HQXXD on CVA. Our previous meta-analysis showed that the total effective rate of HQXXD in the treatment of CVA was not significant compared with the control group [9]. We searched the main database and screened RCTs investigating the clinical curative effect of HQXXD in CVA to perform a meta-analysis, which indicated that the total effective rate of the clinical curative effect was significantly higher in the experimental group than in the control group. Thus, an evidence-based medicine approach was used to indicate the efficacy of HQXXD in CVA from a clinical level, which was more meaningful than experimental validation.

## 5. Conclusion

In conclusion, based on a network pharmacology approach, this study investigated the relationships between the active compounds, target genes, and pathways, and further studied the multicomponent, multitarget, and multipathway characteristics of HQXXD acting on CVA. HQXXD acts on CVA via the effect of multiple compounds related to multiple targets and through multiple pathways. Finally, we investigated the clinical curative effect of HQXXD on CVA using an evidence-based medicine approach, which indicated that the total effective rate of the clinical curative effect was significantly higher in the experimental group compared with the control group. In brief, the pharmacological mechanism of HQXXD acting on CVA has been investigated, and the clinical effect of HQXXD on CVA is remarkable.

## Figures and Tables

**Table 1:** Basic information on the active compounds in HQXXD.

Medicine	Active compound	Compound ID	OB (%)	DL
RA	Mairin	MOL000211	55.38	0.78
RA	Jaranol	MOL000239	50.83	0.29
RA	Hederagenin	MOL000296	36.91	0.75
RA	(3S,8S,9S,10R,13R,14S,17R)-10,13-Dimethyl-17-[(2R,5S)-5-propan-2-yloctan-2-yl]-2,3,4,7,8,9,11,12,14,15,16,17-dodecahydro-1H-cyclopenta[a]phenanthren-3-ol	MOL000033	36.23	0.78
RA	Isorhamnetin	MOL000354	49.6	0.31
RA	3,9-Di-O-methylnissolin	MOL000371	53.74	0.48
RA	5′-Hydroxyiso-muronulatol-2′,5′-di-O-glucoside	MOL000374	41.72	0.69
RA	7-O-Methylisomucronulatol	MOL000378	74.69	0.3
RA	9,10-Dimethoxypterocarpan-3-O-*β*-D-glucoside	MOL000379	36.74	0.92
RA	(6aR,11aR)-9,10-Dimethoxy-6a,11a-dihydro-6H-benzofurano[s3,2-c]chromen-3-ol	MOL000380	64.26	0.42
RA	Bifendate	MOL000387	31.1	0.67
RA	Formononetin	MOL000392	69.67	0.21
RA	Isoflavanone	MOL000398	109.99	0.3
RA	Calycosin	MOL000417	47.75	0.24
RA HRA	Kaempferol	MOL000422	41.88	0.24
RA	FA	MOL000433	68.96	0.71
RA	(3R)-3-(2-Hydroxy-3,4-dimethoxyphenyl)chroman-7-ol	MOL000438	67.67	0.26
RA	Isomucronulatol-7,2′-di-O-glucosiole	MOL000439	49.28	0.62
RA	1,7-Dihydroxy-3,9-dimethoxypterocarpene	MOL000442	39.05	0.48
RA HS	Quercetin	MOL000098	46.43	0.28
HRA	4,9-Dimethoxy-1-vinyl-b-carboline	MOL012140	65.3	0.19
HRA	Caribine	MOL012141	37.06	0.83
HRA	Cryptopin	MOL001460	78.74	0.72
HRA	Sesamin	MOL001558	56.55	0.83
HRA	[(1S)-3-[(E)-But-2-enyl]-2-methyl-4-oxo-1-cyclopent-2-enyl](1R,3R)-3-[(E)-3-methoxy-2-methyl-3-oxoprop-1-enyl]-2,2-dimethylcyclopropane-1-carboxylate	MOL002501	62.52	0.31
HRA	(3S)-7-Hydroxy-3-(2,3,4-trimethoxyphenyl) chroman-4-one	MOL002962	48.23	0.33
HRA	ZINC05223929	MOL009849	31.57	0.83
HS	Schizonepetoside B	MOL011849	31.02	0.28
HS	Schkuhrin I	MOL011856	54.45	0.52
HS	Diosmetin	MOL002881	31.14	0.27
HS RS	Sitosterol	MOL000359	36.91	0.75
HS	5,7-Dihydroxy-2-(3-hydroxy-4-methoxyphenyl)chroman-4-one	MOL005100	47.74	0.27
HS	Luteolin	MOL000006	36.16	0.25
HS RS	Beta-sitosterol	MOL000358	36.91	0.75
HS	Stigmasterol	MOL000449	43.83	0.76
HS	Supraene	MOL001506	33.55	0.42
HS	Campest-5-en-3beta-ol	MOL005043	37.58	0.71
RS	(2R,3R)-3-(4-Hydroxy-3-methoxy-phenyl)-5-methoxy-2-methylol-2,3-dihydropyrano [5,6-h][1,4]benzodioxin-9-one	MOL000011	68.83	0.66
RS	11-Hydroxy-sec-o-beta-d-glucosylhamaudol_qt	MOL011730	50.24	0.27
RS	Anomalin	MOL011732	59.65	0.66
RS	Divaricatacid	MOL011737	87	0.32
RS	Divaricatol	MOL011740	31.65	0.38
RS	Ammidin	MOL001941	34.55	0.22
RS	Ledebouriellol	MOL011747	32.05	0.51
RS	Phelloptorin	MOL011749	43.39	0.28
RS	5-O-Methylvisamminol	MOL011753	37.99	0.25
RS	Phellopterin	MOL002644	40.19	0.28
RS	Wogonin	MOL000173	30.68	0.23
RS	Mandenol	MOL001494	42	0.19
RS	Isoimperatorin	MOL001942	45.46	0.23
RS	Prangenidin	MOL003588	36.31	0.22
RS	Methyl icosa-11,14-dienoate	MOL007514	39.67	0.23
RS	Decursin	MOL013077	39.27	0.38

**Table 2:** Gene symbol and Entrez ID of candidate target genes.

Gene symbol	Entrez ID
PTGER3	5733
MMP2	4313
SLC6A2	6530
ADRA2C	152
HSPB1	3315
PLAU	5328
NOS2	4843
CCNB1	891
SLC6A4	6532
IRF1	3659
UGT1A1	54658
ALOX5	240
GJA1	2697
COL1A1	1277
BIRC5	332
MAOA	4128
PON1	5444
CHEK1	1111
BCL2	596
ADRA2A	150
CYP1A1	1543
E2F1	1869
HTR3A	3359
CASP8	841
PTGES	9536
CHRM1	1128
PPARG	5468
CRP	1401
GSTP1	2950
CXCL8	3576
SELE	6401
FN1	2335
AHR	196
CHRNA2	1135
NFE2L2	4780
THBD	7056
ECE1	1889
MAPK14	1432
ADRB1	153
RAF1	5894
EGF	1950
IL1A	3552
MPO	4353
ADRA1B	147
PCNA	5111
CHUK	1147
SPP1	6696
PTGS2	5743
SLPI	6590
NCF1	653361
CCND1	595
ESR1	2099
ADRB2	154
POR	5447
VEGFA	7422
MYC	4609
ADRA1D	146
CCNA2	890
ACHE	43
MCL1	4170
CCL2	6347
MMP1	4312
STAT1	6772
IL6	3569
CASP3	836
PARP1	142
COL3A1	1281
KDR	3791
ABCG2	9429
HMOX1	3162
MMP3	4314
PPARA	5465
MAOB	4129
CYP1A2	1544
GSTM1	2944
IL10	3586
MAPK1	5594
PLAT	5327
MDM2	4193
CXCL2	2920
EGFR	1956
NQO1	1728
SOD1	6647
IL2	3558
ERBB3	2065
ERBB2	2064
IFNG	3458
FOS	2353
IL4	3565
OPRD1	4985
G6PD	2539
TOP2A	7153
MAPK8	5599
ICAM1	3383
CAV1	857
BCL2L1	598
CHEK2	11200
RELA	5970
HIF1A	3091
NOS3	4846
RB1	5925
PGR	5241
CHRM2	1129
OPRM1	4988
CXCL10	3627
TYR	7299
SERPINE1	5054
VCAM1	7412
CASP9	842
ADH1B	125
CDKN1A	1026
NCOA2	10499
AKT1	207
IL1B	3553
CHRM3	1131
NFKBIA	4792
IGFBP3	3486
PTGS1	5742
F3	2152
NR1I2	8856
JUN	3725
ESR2	2100
BAX	581
PRKCA	5578
CD40LG	959
CYP3A4	1576
TP63	8626
CYP1B1	1545
PRSS1	5644
LTA4H	4048
MMP9	4318
RXRB	6257

**Table 3:** Key compounds in HQXXD acting on CVA.

Compound ID	Compound name	Degree	Medicine
MOL000098	Quercetin	96	RA HS
MOL000006	Luteolin	41	HS
MOL000422	Kaempferol	37	RA HRA
MOL000173	Wogonin	28	RS
MOL000378	7-O-Methylisomucronulatol	23	RA
MOL000358	Beta-sitosterol	22	HS RS
MOL000392	Formononetin	18	RA
MOL000371	3,9-Di-O-methylnissolin	17	RA
MOL000449	Stigmasterol	17	HS
MOL000354	Isorhamnetin	16	RA
MOL000380	(6aR,11aR)-9,10-Dimethoxy-6a,11a-dihydro-6H-benzofurano[3,2-c]chromen-3-ol	14	RA
MOL000417	Calycosin	13	RA
MOL001460	Cryptopin	13	HRA
MOL002962	(3S)-7-Hydroxy-3-(2,3,4-trimethoxyphenyl) chroman-4-one	13	HRA

**Table 4:** Key targets of HQXXD acting on CVA.

Key target	Degree
AKT1	93
IL6	91
VEGFA	89
JUN	81
EGFR	79
CXCL8	79
CASP3	78
MMP9	77
PTGS2	77
MAPK8	76
MAPK1	76
EGF	73
MYC	73
FOS	72
IL1B	72
ESR1	71
CCND1	69
CCL2	66
FN1	64
IL10	63

**Table 5:** Summary of RCTs investigating HQXXD in CVA.

Study year [ref]	Country	Sample size (experimental/control)	Mean age (years) (experimental/control)	Experimental	Control
Wang and Xie 2009 [20]	China	42 (21/21)	34.5 ± 2.7/33.2 ± 2.6	HQXXD	Terbutaline tablet
Fan and Xie 2014 [19]	China	30 (30/30)	42.10 ± 8.27/38.2 ± 10.35	HQXXD	Procaterol hydrochloride tablet and albuterol aerosol
Li 2015 [17]	China	70 (35/35)	50 ± 1.5/49 ± 1.3	HQXXD	Procaterol hydrochloride tablet and albuterol aerosol
Wang and Xie 2015 [18]	China	90 (45/45)	41.12 ± 7.14/40.18 ± 9.35	HQXXD	Procaterol hydrochloride tablet
Wei and Qin 2019 [16]	China	80 (40/40)	45.35 ± 3.88/43.25 ± 3.78	HQXXD	Ambroxol hydrochloride tablet and terbutaline sulfate tablet

## Data Availability

The data used to support this study are included within the supplementary files.

## Supplementary Materials

Supplementary Materials Supplementary file 1: compounds of HQXXD in TCMSP. (A) 87 compounds of RA in TCMSP; (B) 192 compounds of HRA in TCMSP; (C) 159 compounds of HS in TCMSP; (D) 173 compounds of RS in TCMSP. Supplementary file 2: target genes of HQXXD: (A) corresponding target genes of active compounds; (B) corresponding target gene symbols of active compounds; (C) 217 active target genes of HQXXD. Supplementary file 3: CVA-related target genes. (A) CVA-related target genes from GeneCards; (B) 1481 CVA-related target genes. Supplementary file 4: data of the medicine-compound-target network. Supplementary file 5: data of the PPI network. Supplementary file 6. GO function enrichment and KEGG pathway enrichment analysis. (A) BP function enrichment analysis; (B) MF function enrichment analysis; (C) CC function enrichment analysis; (D) KEGG pathway enrichment analysis.
