# Supplementary material for: Network Pharmacology Strategy to Investigate the Pharmacological Mechanism of HuangQiXiXin Decoction on Cough Variant Asthma and Evidence-Based Medicine Approach Validation
Source: Evid Based Complement Alternat Med. 2020 Oct 30;2020:3829092. doi: 10.1155/2020/3829092 (PMC7647767; doi:10.1155/2020/3829092)
Supplement: Supplementary Materials — Supplementary file 1: compounds of HQXXD in TCMSP. (A) 87 compounds of RA in TCMSP; (B) 192 compounds of HRA in TCMSP; (C) 159 compounds of HS in TCMSP; (D) 173 compounds of RS in TCMSP. Supplementary file 2: target genes of HQXXD: (A) corresponding target genes of active compounds; (B) corresponding target gene symbols of active compounds; (C) 217 active target genes of HQXXD. Supplementary file 3: CVA-related target genes. (A) CVA-related target genes from GeneCards; (B) 1481 CVA-related target genes. Supplementary file 4: data of the medicine-compound-target network. Supplementary file 5: data of the PPI network. Supplementary file 6. GO function enrichment and KEGG pathway enrichment analysis. (A) BP function enrichment analysis; (B) MF function enrichment analysis; (C) CC function enrichment analysis; (D) KEGG pathway enrichment analysis. [file 3829092.f1.zip › 3829092.f1/Appendix File Legends.docx]

**Appendix File Legends**

**Appendix File 1 Compounds of HQXXD in TCMSP**

(A) 87 compounds of RA in TCMSP; (B) 192 compounds of HRA in TCMSP; (C) 159 compounds of HS in TCMSP; (D) 173 compounds of RS in TCMSP

**Appendix File 2 Target genes of HQXXD**

(A) Corresponding target genes of active compounds; (B) Corresponding target gene symbols of active compounds; (C) 217 active target genes of HQXXD.

**Appendix File 3 CVA related target genes**

(A) CVA related target genes from GeneCards; (B) 1481 CVA related target genes.

**Appendix File 4 Data of medicine-compound-target network**

**Appendix File 5 Date of PPI network**

**Appendix File 6 GO function enrichment and KEGG pathway enrichment analysis**

(A) BP function enrichment analysis; (B) MF function enrichment analysis; (C) CC function enrichment analysis; (D) KEGG pathway enrichment analysis.
